# Biological and neurocognitive correlates of comorbid post-traumatic stress disorder and alcohol use disorder: a systematic review

**DOI:** 10.1080/20008066.2026.2640736

**Published:** 2026-05-26

**Authors:** Ellen E. Towers, Joel Hoffman, Eva E. Louie, Gezelle Dali, Warren Logge, Katherine Mills, Kirsten C. Morley

**Affiliations:** a Edith Collins Centre for Translational Research in Alcohol, Drugs and Toxicology, Royal Prince Alfred Hospital, Sydney, Australia; b Faculty of Medicine and Health, Specialty of Addiction Medicine, Central Clinical School, University of Sydney, Sydney, Australia; c Faculty of Medicine, Dentistry and Health Sciences, Melbourne School of Psychological Sciences, The University of Melbourne, Melbourne, Australia; d Faculty of Medicine and Health, The Matilda Centre, Sydney Medical School, University of Sydney, Sydney, Australia

**Keywords:** Alcohol use disorder, neurocognition, neuroimaging, molecular, post-traumatic stress disorder, systematic review, Trastorno por uso de alcohol, neurocognición, neuroimagen, molecular, trastorno de estrés postraumático, revisión sistemática

## Abstract

**Background:** Post-traumatic stress disorder (PTSD) and alcohol use disorder (AUD) frequently co-occur, leading to greater clinical burden than either disorder alone. Despite this, little is known about the biological pathways linking these two disorders.

**Objective:** We conducted a systematic review to synthesize evidence on molecular, genetic, neural, and cognitive mechanisms contributing to comorbid PTSD & AUD.

**Method:** Following PRISMA guidelines, we performed a comprehensive search of five databases using PTSD-related, AUD-related, biological, and neurocognitive terms. Participants had to meet diagnostic criteria for both PTSD and AUD, and studies were required to have a comparator including controls, PTSD only, or AUD only. A critical appraisal was completed for all studies.

**Results:** From 3904 identified papers, 14 met the inclusion criteria. Four studies examined the same molecular marker, with three papers derived from the same cohort, investigating baseline and stress-induced cortisol and adrenocorticotropic hormone, and found no differences unique to PTSD & AUD. One study linked low brain-derived neurotrophic factor and hazardous drinking to PTSD onset over 2 years following hospital admission. Genetic studies showed considerable overlap (72%) between PTSD and AUD in female twins, whereby the *DRD2* A1 allele and the absence of the *APOE* ϵ2 allele were strongly associated with PTSD and drinking. Studies also reported lower neurometabolites, white matter integrity, and hippocampal volume in PTSD & AUD. Critical appraisal of these studies highlighted prominent selection bias (predominantly male and veterans) and limited justification of sample size.

**Conclusions:** These findings suggest that PTSD & AUD may be characterized by distinct neurobiological alterations and genetic vulnerabilities relative to comparator groups. However, as there are currently insufficient data to support or refute these findings, this highlights the need for further research.

## HIGHLIGHTS

- PTSD and AUD frequently co-occur, leading to greater clinical burden than either disorder alone. Despite this, little is known about the biological pathways linking these two disorders.
- The current study aimed to review the existing molecular, genetic, neural, and cognitive evidence, and suggests similar impacted stress regulation across PTSD-only, AUD-only, and PTSD & AUD, yet distinct neural and genetic substrates among individuals with PTSD & AUD.
- Future research should include more female participants and a broader range of trauma types (currently dominated by veteran samples), and use large-scale neuroimaging and genetic datasets to help to identify mechanisms underpinning PTSD & AUD.

## Abstract (es)

**Antecedentes:** El TEPT y el TUA frecuentemente coocurren, lo que lleva a una carga clínica más grande que cualquiera de los trastornos en forma separada. A pesar de esto, poco se sabe acerca de las vías biológicas que se relacionan con estos dos trastornos.

**Objetivo:** Se condujo una revisión sistemática para sintetizar la evidencia sobre los mecanismos moleculares, genéticos, neurales y cognitivos que contribuyen a la comorbilidad entre el TEPT y el TUA.

**Método:** Siguiendo las guías PRISMA, se realizó una búsqueda exhaustiva en cinco bases de datos utilizando términos relacionados con el TEPT, relacionados con el TUA, biológicos y neurocognitivos. Los participantes tenían que reunir los criterios diagnósticos tanto para el TEPT y el TUA, y los estudios requerían tener un comparador, incluido controles, solo TEPT o solo TUA. Se completó una evaluación crítica para todos los estudios.

**Resultados:** De los 3.904 artículos identificados, catorce reunían los criterios de inclusión. Cuatro estudios examinaron el mismo marcador molecular, con tres artículos derivados de la misma cohorte, investigando el cortisol y la hormona adrenocortical al inicio e inducidos por estrés y no hubo diferencias únicas para el TEPT y el TUA. Un estudio asoció bajos niveles del factor neurotrófico derivado del cerebro y consumo riesgoso de alcohol al inicio del TEPT a dos años de seguimiento tras una hospitalización. Los estudios genéticos demostraron una superposición importante (72%) entre el TEPT y el TUA en gemelares mujeres, donde el alelo *DRD2* A1 y la ausencia del alelo *APOE* ϵ2 se asociaron fuertemente con el TEPT y consumo de alcohol. Los estudios también reportaron niveles más bajos de neurometabolitos, de la integridad de la sustancia blanca, y del volumen hipocampal en el TEPT y el TUA. La evaluación crítica de estos estudios destaca el sesgo de selección prominente (predominantemente varones y veteranos) y una justificación limitada del tamaño de la muestra.

**Conclusiones:** Estos hallazgos sugieren que el TEPT y el TUA se pueden caracterizar por alteraciones neurobiológicas distintas y vulnerabilidades genéticas relativas al grupo comparador. Sin embargo, como actualmente no hay suficientes datos para apoyar o refutar estos hallazgos, esto enfatiza la necesidad de investigaciones futuras.

## 1. Introduction

Co-occurring post-traumatic stress disorder (PTSD) and alcohol use disorder (AUD) (PTSD & AUD) is a highly prevalent comorbidity in clinical (Bonin et al., 2000; Brown et al., 1999; Gielen et al., 2012; Reynolds et al., 2005; Triffleman et al., 1995) and community settings (Alonso et al., 2004; Creamer et al., 2001; Grant et al., 2015; Kessler et al., 1995; Longo et al., 2020). Importantly, people with PTSD & AUD have significantly worse functional, prognostic, and social sequelae than those with either disorder alone (Blanco et al., 2013; Goldstein et al., 2016; Norman et al., 2018; Read et al., 2004). Despite this, there is limited research examining the biological basis of this comorbidity. The biology underpinning these disorders has historically been studied in isolation (Gilpin & Weiner, 2017; Suh & Ressler, 2018), which ignores how these biological processes occur and interact on an individual level. A synthesis of the current research on the biological and neurocognitive mechanisms of PTSD & AUD is required to help to delineate key gaps in our knowledge.

The strong association between PTSD and AUD has been established for several decades through numerous clinical and epidemiological studies (Alonso et al., 2004; Creamer et al., 2001; Kessler et al., 1995; Scherrer et al., 2008). Despite changing clinical definitions of each condition, this comorbidity remains one of the most common neuropsychiatric dual diagnoses (Smith & Cottler, 2018). In Australia, the probability of having PTSD given AUD is 12.1%, and the probability of AUD given PTSD 6.4% (Sunderland et al., 2025). In line with this, other national epidemiological studies suggest that 15–30% of those with AUD will also meet criteria for PTSD (Castillo-Carniglia et al., 2019; Preuss et al., 2018). These prevalence patterns are concerning, as people with PTSD & AUD have significantly worse functioning than those with either disorder alone. That is, individuals with PTSD & AUD demonstrate greater symptom severity (Blanco et al., 2013; Goldstein et al., 2016; Longo et al., 2020; McCarthy & Petrakis, 2010), higher rates of additional psychiatric comorbidities (Goldstein et al., 2016; Norman et al., 2018), and greater physical health and psychosocial problems (Evren et al., 2011; Forbes et al., 2015; Norman et al., 2018). Together, these findings underscore the need to clarify the mechanisms driving PTSD & AUD comorbidity and determine whether they arise from additive effects of each disorder or from distinct pathways.

Multiple biological interacting mechanisms have been identified as likely contributors to PTSD & AUD. The hypothalamic–pituitary–adrenal (HPA) axis is the primary neuroendocrine system that responds to stress (McEwen et al., 2015), and is frequently cited in the PTSD & AUD literature (Gilpin & Weiner, 2017; Suh & Ressler, 2018). In the independent conditions there is a complex dysregulation of this system; for example, individuals with AUD show elevated cortisol during intoxication (Adinoff et al., 2003; Kutscher et al., 2002), but a blunted response during early abstinence (Bernardy et al., 1996; Errico et al., 1993; Sinha et al., 2009), whereas PTSD is typically associated with lower cortisol compared to both trauma-exposed controls (TECs) (Yehuda et al., 1995) and trauma-naïve controls (TNCs) (Metz et al., 2020; Morris et al., 2012). Beyond the direct impact of alcohol and stress on the HPA axis, it is likely that genetic factors are also implicated in each of these conditions. For example, the *FKBP5* gene regulates glucocorticoid receptor function, and has been linked to PTSD risk in the context of trauma exposure (Hawn et al., 2019) and drinking (mediated by early life stress) (Dragan et al., 2018; Nylander et al., 2017). Preclinical evidence suggests that *FKBP5* gene inhibitors reduce stress-induced drinking and fear responses (Cruz et al., 2023). Dysregulation of the HPA axis also has downstream effects on the function of many brain regions, such as the amygdala and hippocampus, through glucocorticoid receptor signalling (see review by Myers et al., 2014). These regions have been extensively studied in each condition independently (Logue et al., 2018; Mackey et al., 2019; Wrase et al., 2008) and regarding their comorbidity (Schuff et al., 2008; Woodward et al., 2006), and converging evidence highlights amygdala and hippocampal function as being necessary for learning, memory, and emotional regulation (for reviews, see Eichenbaum, 2004; LeDoux, 2000; Squire, 1992; see also Wu et al., 2025). Accordingly, although much research highlights mechanistic overlap between the two conditions, clarifying whether there are distinct mechanisms underpinning this comorbidity remains essential to advancing our knowledge.

To date, several reviews have examined the biological mechanisms of comorbid PTSD and substance use disorder (Carlson & Weiner, 2021; Gilpin & Weiner, 2017; Hien et al., 2021; Hruska & Delahanty, 2014; Norman et al., 2012; Suh et al., 2019), but this is the first to systematically evaluate these mechanisms specifically in PTSD & AUD. Accordingly, the goal of the present study is to provide a systematic review of the available evidence on biological and neurocognitive mechanisms contributing to PTSD and AUD comorbidity, aiming to inform future research and treatment development.

## 2. Method

This review follows the Preferred Reporting Items for Systematic Reviews and Meta-Analyses (PRISMA) guidelines for systematic reviews (Moher et al., 2009; Shamseer et al., 2015). Before starting the database search, we searched within the International Prospective Register of Systematic Reviews (PROSPERO) database for possible registered protocols that may have been conducting the same systematic review. No such protocol was identified on the database; thus, a protocol was registered on PROSPERO (registration number: 42024556983 available from URL https://www.crd.york.ac.uk/prospero/display_record.php?ID=CRD42024556983). Any deviations from the registered protocol were logged and are available on PROSPERO.

### 2.1. Search strategy

To ensure a comprehensive review, the search strategy was conducted using the following databases: Embase, MEDLINE via Ovid, Scopus, APA PsycINFO, and Web of Science.

The search strategy incorporated terms relating to PTSD, AUD (including heavy/hazardous drinking) and terms relating to biological and neurocognitive markers. For specific search strategy details for each database, see Supplementary Methods. These search terms were a mix of medical subject headings (MeSH) (where permitted) and relevant key words. Keywords often used truncation operators. Within each search section, MeSH terms and keywords were combined using the Boolean operator ‘OR', while different sections were combined using the Boolean operator ‘AND’. The search strategy for each database is detailed in Supplementary Methods.

### 2.2. Study selection

Once all the database searches had been completed and duplicate studies removed, a multi-stage screening process was performed by four authors (ET, GD, WL, EL). Studies were screened in the following order: (1) title and abstracts, and (2) full-text article. Titles and abstracts were screened to ensure that studies included individuals with PTSD and AUD (or heavy/hazardous drinking), and that biological outcomes were included. In the final stage, all remaining studies underwent a full-length text review. All full-text studies had to satisfy the eligibility criteria outlined in Section 2.3.

### 2.3. Eligibility criteria

To be eligible for the current review, studies were required to examine individuals with current PTSD and AUD (or heavy/hazardous drinking) relative to a comparator group. Lifetime diagnoses of PTSD and AUD were permissible for genetic studies. A comparator group could be any of the following: PTSD only, AUD only (including heavy/hazardous drinking), trauma-exposed controls (TECs), or trauma-naïve controls (TNCs). Heavy/hazardous drinking was defined as National Institute of Alcohol Abuse and Alcoholism (NIAAA) standards or World Health Organization (WHO) guidelines. Specifically, the NIAAA defines heavy drinking as consuming at least five standard drinks per drinking day for males and at least four for females, while hazardous drinking was assessed by the WHO guidelines Alcohol Use Disorders Identification Test (AUDIT), scoring ≥ 8 (Saunders et al., 1993). As such, diagnoses of AUD or alcohol dependence, as well as meeting WHO or NIAAA thresholds for hazardous or heavy drinking, were collectively operationalized as AUD in this review. PTSD was required to be diagnosed by a practitioner, and not by self-report, owing to notable discrepancies between these methods (Blanchard et al., 1996; Forbes et al., 2001; Kramer et al., 2023; Moshier et al., 2018). Studies were excluded if participants presented with additional psychiatric, substance use disorder, or physical health comorbidities. Studies were excluded if they were post-mortem or animal investigations, or if they only investigated treatment effects or did not investigate the comorbidity itself. No restrictions on language or publication date were applied to the database searches, but only English-language articles were included. Secondary data sources, such as systematic reviews or meta-analyses, were excluded. Additionally, dissertations, conference proceedings, book chapters, editorials, unpublished studies, case studies, and other grey literature were not included. To ensure comprehensive coverage, we manually searched the reference lists of relevant systematic reviews and all studies for which the full text was retrieved, aiming to identify any additional studies.

### 2.4. Data extraction

Three reviewers (EET, GD, JH) used a customized form to extract study information essential for evaluating the relevant data. The extracted data included several key elements. First, general study details included (1) study country, (2) cohort characteristics (e.g. sample size, mean age with standard deviation, sex distribution, and target population), (3) details on study inclusion and exclusion criteria, (4) study design, (5) comparator cohorts, and (6) diagnostic criteria. For all studies, methodological details, results, and conclusions were gathered, including (1) adjustments for bias (covariates), (2) PTSD severity, (3) alcohol outcomes and consumption in standard units, (4) missing data, (5) smoking status, (6) inclusion of additional psychiatric measures, (7) statistical test used, (8) primary and secondary outcomes, (9) conclusions, and (10) limitations. For each specific biomarker, we extracted data specific to the domain (see further details in Supplementary Methods). A customized data extraction file is also available in the Supplementary material.

### 2.5. Quality assessment

The methodological quality of the studies was assessed with the Appraisal tool for Cross-Sectional Studies (AXIS) (Downes et al., 2016) and Newcastle–Ottawa Scale (NOS) for any longitudinal studies (Wells et al., 2000). Two authors (ET, EL) independently evaluated each study, and disagreements were solved by a third author (GD or JH). For each item of the assessment tool, a score of 1 was assigned if the study satisfied the items in terms of methodological adequacy; otherwise, the assigned score was 0. For each paper, the number of items with a score of 1 is reported, providing an estimate of the overall methodological quality of the study.

## 3. Results

**Figure 1.**
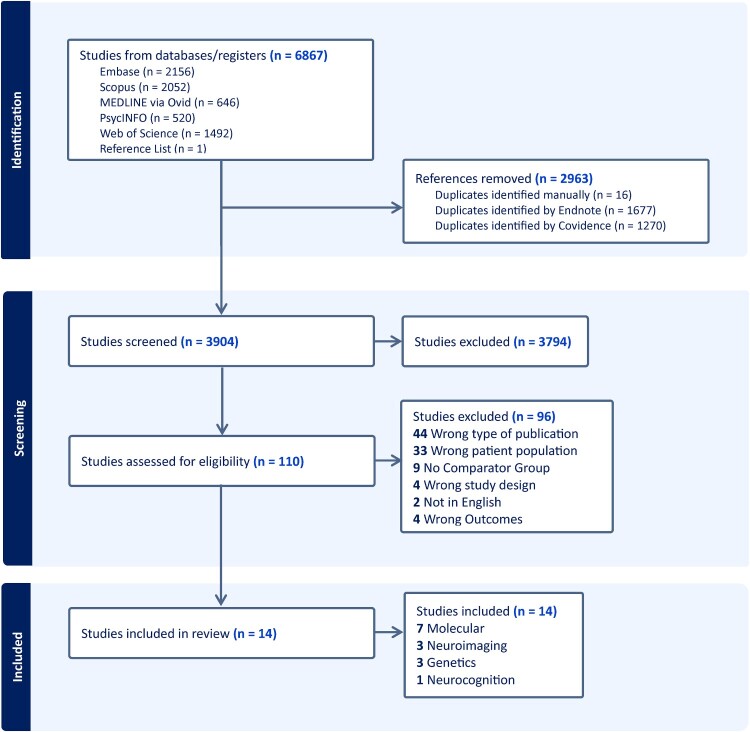
Preferred Reporting Items for Systematic Reviews and Meta-Analyses (PRISMA) flowchart of the study selection process. Search strategy completed on 20 November 2024; search repeated on 30 October 2025 (data not presented here), no new papers included.

The primary search identified 6866 studies: 2156 from Embase, 2052 from Scopus, 646 from MEDLINE via Ovid, 520 from PsycINFO, and 1492 from Web of Science. Duplicates were removed (*n* = 2963), and the remaining papers were screened according to the eligibility criteria outlined in Section 2.3. Titles and abstracts were screened, and 3794 studies were excluded, leaving 110 studies for full-text screening. Articles were reviewed against the eligibility criteria and 96 were excluded at this stage. The main reasons for exclusion were wrong type of publication (i.e. from conference proceedings), wrong patient population, and no adequate comparator group (Figure 1). A manual search of the reference lists of the 14 selected studies revealed that one study was potentially appropriate for this review, but no full text was available in English and therefore it was not included in the review (Thaller et al., 2003). The primary search was conducted on 20 November 2024, and the search was repeated on 30 October 2025, at which point no additional papers were identified.

### 3.1. Study characteristics

**Figure 2.**
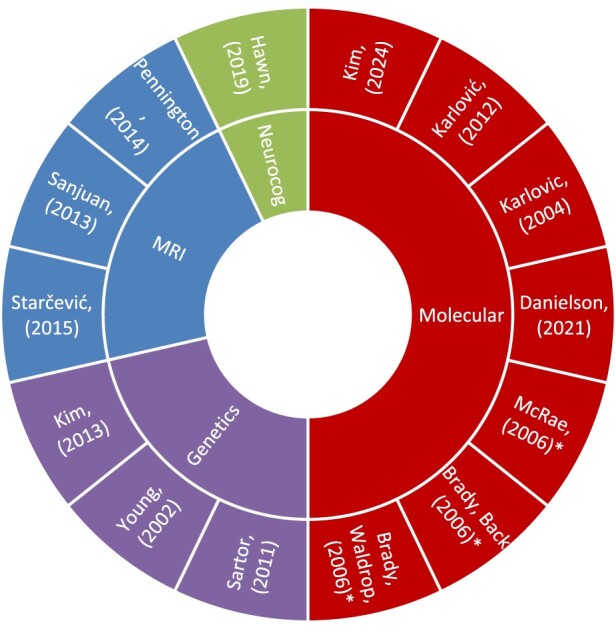
Sunburst chart. The proportion of studies using each biomarker domain is shown in the inner ring. *Same participant sample. Neurocog = neurocognition; MRI = magnetic resonance imaging.

Fourteen studies fulfilled the eligibility criteria for the study. These studies were published between 2002 and 2024. All participants with PTSD were clinically diagnosed by a professional [using the Diagnostic and Statistical Manual of Mental Disorders, 4th Edition (DSM-IV), 5th Edition (DSM-5), or International Classification of Diseases, 10th Revision (ICD-10) criteria], while alcohol use was captured by either clinical diagnosis or drinking at a predefined heavy or hazardous level. For more detailed information about alcohol use criteria across the studies, see Table 1. Importantly, three studies (Danielson et al., 2021; Hawn et al., 2019; Kim et al., 2024) explicitly excluded individuals with current alcohol dependence (assessed by the DSM-IV), and one study was investigating lifetime AUD diagnosis (Sanjuan et al., 2013); however, these studies were included as those with PTSD were engaging in heavy/hazardous drinking. The variety of alcohol-related criteria will henceforth be referred to broadly as AUD. Furthermore, a variety of comparator groups was included across these 14 studies, as outlined in Table 1. Population characteristics and a summary of findings for each study are also provided in Table 1. Moreover, there were three papers that used the same cohort (Brady, Back, et al., 2006; Brady, Waldrop, et al., 2006; McRae et al., 2006), which are depicted by the categorizations of the outer ring of the sunburst plot (Figure 2).

**Table 1.** Study characteristics of included studies.

Study	Comparator groups	Sex (% F)	Population	PTSD diagnosis	AUD definition	Findings	
						Significant	Not significant
Molecular							
Danielson et al. (2021)	36 TNC	58.3	Intimate partner violence	CAPS-IV	TLFB^d^		Across all groups: baseline cortisol; cortisol reactivity to TSST
	35 TEC^a^	60					
	27 PTSD	59.3					
Brady, Back, et al. (2006)^e^	34 AUD	45.7	Community	CAPS-IV	SCID-IV	In AUD: low ACTH response with greater craving increase after CPT did not predict higher follow-up drinking	In PTSD & AUD: low ACTH response with greater craving increase after CPT did not predict higher follow-up drinking
	28 PTSD & AUD	42.9					
McRae et al. (2006)^e^	11 PTSD & AUD	45	Community	CAPS-IV	SCID-IV		Across groups: (ACTH)/cortisol group differences in CPT or TSST
	10 AUD						
	4 PTSD						
	6 CON^b^						
Brady, Waldrop, et al. (2006) ^e^	28 PTSD & AUD	43	Community	CAPS-IV	SCID-IV	Compared to AUD or CON: in PTSD & AUD: slower cortisol recovery from CPT Compared to CON: in PTSD & AUD: lower ACTH response to CPT	Across groups: peak cortisol or ACTH response to CPT
	31 AUD	44					
	30 PTSD	60					
	30 CON^b^	50					
Karlović et al. (2004)	43 PTSD	0	Veterans	DSM-IV	CAGE & SCID-IV	Compared to CON: higher TT3 in PTSD & AUD	PTSD vs PTSD & AUD: TT3 Across all groups: FT3, TT4, FT4, and TSH
	41 PTSD & AUD						
	39 CON^b,c^						
Karlović et al. (2012)	17 PTSD	0	Veterans	CAPS-IV	CAGE & SCID-IV PTSD & AUD abstinent for 4–10 weeks	Compared to PTSD: higher basal testosterone in PTSD & AUD	PTSD & MDD vs CON vs PTSD & AUD: basal testosterone
	29 PTSD & AUD						
	18 PTSD & MDD						
	34 TEC						
Kim et al. (2024)	Non-drinker at BL = 441	46.5	Patients hospitalized following severe physical injury	CAPS-5	AUDIT	Compared to non-HD: lower serum BDNF at injury predicted PTSD at 1 and 2 year follow-up only in HD	
	Current drinker at BL = 482	11.6					
Genetic							
Sartor et al. (2011)	46 PTSD & AUD 92 PTSD 409 AUD 3209 CON^b^	100	Community (female–female twins)	DSM-IV	DSM-IV	Genetic correlation between PTSD and AUD was 0.54	
Young et al. (2002)	38 PTSD & AUD	0	Veterans	DSM-IV	≥ 60 g/day	Compared to PTSD & TNC: higher *DRD2* A1 allele frequency in PTSD & AUD	
	53 PTSD	0					
	51 CON^b,c^	65					
Kim et al. (2013)	128 PTSD (46.9% harmful drinking)	0	Korean veterans	CAPS-IV	AUDIT (cut-off 12)	*APOE* ϵ2 non-carriers and harmful drinking interact to increase PTSD risk	*APOE* ϵ2 carrier status and harmful drinking in predicting PTSD risk
	128 TEC (28.9% harmful drinking)	0					
Neurocognition							
Hawn et al. (2019)	302; 99 met criteria for PTSD	11.5	OEF/OIF/OND veterans	CAPS-IV	TLFB^d^		Neither NU nor RTP moderated the relationship between PTSD and drinking
Neuroimaging							
Pennington et al. (2014)	10 PTSD & AUD	0	Trauma-exposed veterans or civilians	CAPS-IV	AUDIT	Compared to TEC and PTSD: lower in PTSD & AUD: Glu and MI in ACC; NAA in TEMP Compared to PTSD: lower in PTSD & AUD: Glu in TEMP	Across groups: NAA, Cr, Cho, MI, Glu, GABA in POC Compared to TEC: Glu in TEMP in PTSD & AUD
	28 PTSD						
	20 TEC						
Sanjuan et al. (2013)	12 PTSD & AUD 10 lifetime AUD	0	Veterans (Afghanistan and Iraq)	CAPS-IV	DSM-IV (lifetime)	Compared to AUD: lower FA bilateral dorsal cingulum and right anterior corona radiata; lower AD left dorsal cingulum	
Starčević et al. (2015)	29 PTSD & AUD	0	Inpatients for PTSD at hospital	ICD-10	ICD-10	Compared to PTSD and CON: lower left and right hippocampus volume in PTSD & AUD	Across groups: amygdala, prefrontal cortex, or ICV
	25 PTSD	0					
	25 CON ^b^	0					

Note: ^a^ Engaging in heavy drinking. ^b^ Control group where trauma exposure status was not specified, or sample did not distinguish between TEC and TNC. ^c^ Population recruited from the community. ^d^ Excluded alcohol dependence based on DSM-IV. ^e^Papers are part of the same study/cohort.

ACC = anterior cingulate cortex; ACTH = adrenocorticotropic hormone; AD = axial diffusivity; *APOE* = apolipoprotein E; AUD = alcohol use disorder; AUDIT = Alcohol Use Disorder Identification Test; BDNF = brain-derived neurotrophic factor; BL = baseline; CAPS-IV = Clinician-Administered PTSD Scale for DSM-IV; CAPS-5 = Clinician-Administered PTSD Scale for DSM-5; Cho = choline; CON = control group; CPT = cold pressor test; *DRD2* = dopamine receptor D_2_; DSM-IV = Diagnostic and Statistical Manual of Mental Disorders, 4th Edition; FA = fractional anisotropy; FT3 = free triiodothyronine; FT4 = free thyroxine; GABA = gamma-aminobutyric acid; Glu = glutamate; ICD-10 = International Classification of Diseases, 10th Revision; ICV = intracranial volume; MDD = major depressive disorder; MI = myoinositol; NAA = *N*-acetylaspartate; NU = negative urgency; OEF = Operation Enduring Freedom; OIF = Operation Iraqi Freedom; OND = Operation New Dawn; PTSD = post-traumatic stress disorder; RTP = risk-taking propensity; SCID-IV = Structured Clinical Interview for DSM-IV; TEC = trauma-exposed control; TEMP = lateral temporal cortex; TLFB = timeline follow-back; TNC = trauma-naïve control; TSH = thyroid-stimulating hormone; TSST = Trier Social Stress Test; TT3 = total triiodothyronine; TT4 = total thyroxine.

#### 3.1.1. Molecular correlates

Molecular correlates were the most investigated domain, examined in seven of the 14 studies. Three of these drew on the same cohort but were published separately (Brady, Back, et al., 2006; Brady, Waldrop, et al., 2006; McRae et al., 2006). Stress-related hormones were the focus of four studies (Brady, Back, et al., 2006; Brady, Waldrop, et al., 2006; Danielson et al., 2021; McRae et al., 2006). Other markers were each examined by a single study, including brain-derived neurotrophic factor (BDNF) (Kim et al., 2024), thyroid hormones (Karlović et al., 2004), and testosterone (Karlović et al., 2012). Findings from each molecular study are summarized in the following subsections, with methodological details presented in Table 2.

**Table 2.** Collection methods and analysis for molecular studie*s.*

Study	Hormone	Task	Procedure and collection	Measurement method
Brady, Back, et al. (2006)	ACTH, cortisol	CPT	Between 7 and 8 a.m., two baseline subjective and blood samples were collected 10 min apart and averaged. Additional samples and ratings were taken immediately before and after CPT, and at 5, 30, 60, and 120 min post-CPT. For CPT, participants were asked to submerge one hand in a cold-water bath (4°C) up to the wrist for as long as they could or for a maximum of 1 min^a^ TSST was conducted at 8 a.m., 2 months after CPT, following a similar procedure. Only included in McRae et al. For TSST, participants were told they would need to give a 5 min impromptu speech to three individuals unknown to them with only 5–10 min to prepare. Following the speech, the participants were asked to perform a 5 min maths challenge to count backwards from 1022 by 13 as quickly and accurately as possible. If an error was made, the subject was instructed to begin again at 1022^a^	Samples collected by qualified nurses in the NIH-supported GCRC ACTH: measured using Allegro HS-ACTH Cortisol: measured with Roche Diagnostics Elecsys 2010 electrochemiluminescence competitive immunoassay [functional sensitivity 8.0 nM/L (0.29 mg/dL); intra-assay CV < 2%]
Brady, Waldrop, et al. (2006)				
McRae et al. (2006)		CPT and TSST		
Danielson et al. (2021)	Cortisol	TSST (or non-stress control condition)	Participants arrived at 4 p.m.; TSST administered at 4:55 p.m. after catheter set-up. Cortisol samples were taken pre-TSST and 25 min post-TSST (10 min after alcohol priming; ∼4:55 p.m., 5:15 p.m., 5:40 p.m., 6:40 p.m.)	Cortisol: measured using Diagnostic Systems Laboratories Cortisol Enzyme Immunoassay Kits (detection limit 0.1 mg/dL; intra- and inter-assay CV < 7%)
Karlović et al. (2004)	TT3, FT3, FT4, TT4, TSH	N/A	Blood sample collected between 8 and	Measured using commercial luminoimmuno
Karlović et al. (2012)	Basal testosterone	N/A	Blood sample collected between 8 and 9 a.m. following a 12 h fast	Measured by radioimmunoassay using commercial kits (Kabi Pharmacia Diagnostics, Uppsala, Sweden; sensitivity 4 ng/dL; interassay CV 5%)
Kim et al. (2024)	Baseline serum BDNF	N/A	Morning samples collected after an overnight fast to minimize effects of stress or activity	BDNF: measured using Quantikine^®^ ELISA Human BDNF Immunoassay (R&D Systems, Minneapolis, USA)

Note: ^a^ The procedure for the CPT and TSST was the same in each research study.

ACTH = adrenocorticotropic hormone; BDNF = brain-derived neurotrophic factor; CPT = cold pressor test; CV = coefficient of variation; ELISA = enzyme-linked immunosorbent assay; FT3 = free triiodothyronine; FT4 = free thyroxine; GCRC = General Clinical Research Center; HS-ACTH = high-sensitivity adrenocorticotropic hormone; N/A = not applicable; TSH = ethyroid-stimulating hormone; TSST = Trier Social Stress Test; TT3 = total triiodothyronine; TT4 = total thyroxine.

##### 3.1.1.1. Cortisol and adrenocorticotropic hormone

Overall, studies did not find significant altered baseline or stress-induced hormone reactivity in PTSD & AUD compared to PTSD or AUD alone. Three studies used the same cohort but employed different analytical techniques. Brady, Waldrop, et al. (2006) examined baseline and stress-induced reactivity in cortisol and adrenocorticotropic hormone (ACTH) across four groups (control, PTSD & AUD, PTSD, and AUD), using the cold pressor test (CPT) (Wirch et al., 2006) (for more details, see Table 2). The study found no differences between groups in baseline cortisol, ACTH, or cortisol stress reactivity, but the control group had a significantly higher ACTH stress reactivity compared to all other groups. Also, individuals with PTSD (PTSD and PTSD & AUD) had a slower return to baseline cortisol than the groups without PTSD (AUD and control) following the CPT. Brady, Back, et al. (2006) investigated the relationship between subjective and neuroendocrine stress reactivity to the CPT and future alcohol use among individuals with AUD or PTSD & AUD. They found that among individuals with AUD, higher cravings combined with a lower ACTH response to the CPT predicted increased future drinking; however, this association was not observed in the PTSD & AUD group. Finally, in McRae et al. (2006), a sample of participants from the overarching study also completed the Trier Social Stress Test (TSST) (Kirschbaum et al., 1993) (for details, see Table 2). The study examined baseline levels and stress reactivity of cortisol and ACTH across PTSD & AUD, PTSD, AUD, and control groups, but found no significant group differences or interactions for either hormone on either task (TSST or CPT).

Danielson et al. (2021) examined how interpersonal violence, stress response, and drinking to cope converge to predict stress-induced drinking, a risk factor for AUD. The TSST was used to induce stress, and administration was the same as in McRae et al. (2006) (for details, see Table 2). There were no associations in the PTSD group between average drinks per drinking day (in the month prior to the study) and baseline cortisol levels or cortisol reactivity to the TSST.

##### 3.1.1.2. BDNF

J. M. Kim et al. (2024) assessed serum BDNF (sBDNF) from patients admitted to a trauma centre and followed them prospectively for 2 years post-admission. At baseline, sBDNF levels and alcohol consumption history were assessed, and PTSD diagnosis was assessed during follow-up (3, 6, 12, and 24 months post-admission). Participants were categorized into two groups based on their alcohol drinking status: non-current drinkers (never drinkers and ex-drinkers) and current drinkers. The authors found no significant associations between Clinician-Administered PTSD Scale for DSM-5 (CAPS-5) total and subscale scores and sBDNF, or between hazardous alcohol use and PTSD. There were no significant associations of sBDNF levels with PTSD in non-drinkers and non-hazardous alcohol users. However, lower sBDNF levels (< 13.7 ng/mL) significantly predicted PTSD in those who currently drank alcohol or drank hazardously, but not for non-drinkers and non-hazardous drinkers. This was only present at later follow-up time-points (12 and 24 months).

##### 3.1.1.3. Thyroid and testosterone hormones

Karlović et al. (2004) investigated differences in thyroid hormone levels in male soldiers with PTSD with and without AUD, recruited from an inpatient treatment centre, versus controls (hospital staff). Serum concentrations of free triiodothyronine (FT3), total triiodothyronine (TT3), free thyroxine (FT4), total thyroxine (TT4), and thyroid-stimulating hormone (TSH) were analysed. Soldiers with PTSD and PTSD & AUD had higher TT3 concentrations compared to the control group, but this did not differ between the PTSD & AUD and PTSD groups. Serum concentrations of FT3, TT4, FT4, and TSH were equal across all three groups.

Karlović et al. (2012) also investigated basal testosterone across those with PTSD, PTSD & AUD, PTSD with concurrent depression (PTSD & DEP), and TEC. In comparison to the TEC group, all participants with PTSD, without considering comorbid conditions, showed no significant differences in basal serum testosterone levels. However, pairwise comparisons within the PTSD cohort revealed that the PTSD-only group had higher basal testosterone concentration than the PTSD & AUD, PTSD & DEP, and TEC groups.

##### 3.1.1.4. Summary

Molecular studies primarily focused on stress hormones and generally found no consistent differences in baseline or stress-induced cortisol and ACTH reactivity across PTSD & AUD, PTSD-only, and AUD-only groups. Lower sBDNF predicted later PTSD only among drinkers; given BDNF’s critical role in neuronal survival and growth (Bathina & Das, 2015), the authors suggest that alcohol may impair BDNF, reducing neuroplasticity and increasing susceptibility to PTSD. Basal testosterone levels were lower in PTSD & AUD compared to PTSD only, a difference not observed relative to TEC or PTSD with depression, indicating a complex relationship in which comorbid conditions such as depression or AUD may mask or modify hormonal alterations. TT3 concentrations were similar between PTSD only and PTSD & AUD but elevated relative to controls; because TT3 can enhance alertness and responsiveness (Armstrong et al., 2023), this increase may contribute to hyperarousal symptoms in PTSD. Overall, molecular evidence for distinct substrates in PTSD & AUD remains limited and inconsistent.

#### 3.1.2. Genetic correlates

Three studies investigated the genetics correlates of PTSD & AUD (Kim et al., 2013; Sartor et al., 2011; Young et al., 2002). Sartor et al. (2011) explored common genetic influences on PTSD and AUD in female twins, whereas Young et al. (2002) and Kim et al. (2013) investigated candidate genes, assessing allele differences between veterans with PTSD, with and without comorbid AUD.

In a large, all-female community sample of twins (*n* = 3768), Sartor et al. (2011) found that over two-thirds of variance in PTSD (71%) and AUD (72%) was attributable to additive genetic effects, with the remainder accounted for by individual-specific environmental influences. The genetic correlation between PTSD and AUD was 0.54. Young et al. (2002) found increased dopamine receptor D_2_ (*DRD2*) A1 allelic frequency in veterans with PTSD, but only in those with concurrent AUD. Also, veterans with PTSD with the A1^+^ (A1A1, A1A2) allele consumed more than twice the amount of alcohol as those with the A1^−^ allele (A2A2). The *DRD2* gene is involved in dopamine functioning (Beaulieu & Gainetdinov, 2011). Kim et al. (2013) found that apolipoprotein E (*APOE*) ϵ2 allele carriers, regardless of drinking status, had an increased risk of developing PTSD. PTSD, in general, was associated with more harmful drinking behaviours, most particularly in individuals who were non-carriers of the *APOE* ϵ2 allele. The absence of the ϵ2 allele and harmful drinking patterns interact to significantly increase PTSD risk, highlighting a potential genetic and behavioural vulnerability. The *APOE* gene is important in stimulating neurite outgrowth in response to cellular injury and degeneration (Huang & Mahley, 2014).

##### 3.1.2.1. Summary

These genetic studies highlight both shared and specific influences in PTSD & AUD. A twin study showed substantial heritability for PTSD and AUD (∼70%) and a strong genetic correlation between them. Candidate gene studies implicated the *DRD2* A1 allele in heightened alcohol use among veterans with PTSD, and the absence of the *APOE* ϵ2 allele was found to increase PTSD risk, particularly among harmful drinkers. This may reflect that a reduced dopaminergic reward function (*DRD2*) or an altered neural repair mechanisms (*APOE*) could increase susceptibility to PTSD following trauma.

#### 3.1.3. Neural correlates

Three studies used imaging modalities to assess the impact of PTSD & AUD on brain structure and function (Pennington et al., 2014; Sanjuan et al., 2013; Starčević et al., 2015), using methods that assessed neurometabolite data, white matter integrity, and grey matter volume. The findings from each study are outlined in the following paragraphs.

First, Pennington et al. (2014) examined neurometabolites from participants recruited from the San Francisco Veterans Affairs Medical Center. Brain metabolite data were evaluated in three volumes of interest: the anterior cingulate, lateral temporal, and parieto-occipital cortices. The neurometabolites examined were *N*-acetylaspartate, creatine, choline-containing compounds, myoinositol, glutamate, and gamma-aminobutyric acid (GABA). In the PTSD & AUD group, glutamate, myoinositol, and choline in the anterior cingulate cortex were lower compared both to the PTSD and TEC groups. There was no significant difference between these neurometabolites in the anterior cingulate cortex between the PTSD and TEC groups. In addition, *N*-acetylaspartate levels in the lateral temporal cortices were lower in the PTSD & AUD group relative to both PTSD and TEC (but not between PTSD and TEC). Finally, glutamate levels in the lateral temporal cortices were normal in PTSD & AUD, but higher in PTSD, relative to TEC. No neurometabolites in the parietal occipital cortices were significantly different between the groups.

Sanjuan, Thoma et al. (2013) examined neural white matter integrity using diffusion tensor imaging (DTI) in combat veterans recruited from the University of New Mexico. DTI data were collected and analysed using a seed-based approach, which included the bilateral dorsal cingulum, parahippocampal cingulum, and anterior corona radiata (ACR). Across these seeds, the DTI metrics that were captured comprised fractional anisotropy (FA), mean diffusivity, axial diffusivity (AD), and radial diffusivity. This study found that individuals with PTSD & AUD had lower FA in the bilateral dorsal cingulum and the right ACR compared to individuals with lifetime AUD only. This effect was similarly present for AD in the left dorsal cingulum in those with PTSD & AUD compared to individuals with lifetime AUD only. The study also found that lower FA in the right ACR was significantly correlated with higher PTSD severity, but not with alcohol use severity.

Starčević et al. (2015) examined structural cortical and subcortical brain differences associated with PTSD & AUD in veterans and healthy controls recruited from the Psychiatric Center for PTSD Treatment in Belgrade, Serbia. T1-weighted magnetic resonance imaging scans were collected and analysed using a region-of-interest approach, including each hemisphere of the hippocampus, lateral prefrontal cortex, anterior cingulate cortex, caudate nucleus, nucleus accumbens, putamen, globus pallidus, thalamus, and the lateral ventricle. Groups were only compared on respective hemispheres of the hippocampus, amygdaloid complexes, prefrontal cortices, total hemisphere volume, and total intracranial brain volume. This study found that the left and right hippocampus had lower volume in the PTSD & AUD group (following Bonferroni correction) compared to the PTSD and control groups. No statistical differences and no correlations were revealed in the amygdala, prefrontal cortex, or total intracranial volume.

##### 3.1.3.1. Summary

Three neuroimaging studies identified convergent brain alterations in individuals with comorbid PTSD and AUD. These findings included reduced neurometabolite levels in the anterior cingulate and temporal cortices, indicating greater metabolic injury in PTSD & AUD relative to PTSD only. In addition, PTSD & AUD was characterized by reduced white matter integrity within limbic–thalamocortical pathways compared with AUD only, suggesting disruption to circuits important for top–down regulatory control. However, without concurrent functional connectivity data, the functional implications of these structural deficits should be interpreted with caution. Finally, evidence indicated that co-occurring AUD exacerbates deterioration of the hippocampal formation in individuals with PTSD. Collectively, these findings point to multimodal neurobiological disruption in comorbid PTSD and AUD.

#### 3.1.4. Neurocognition

One study examined the potential moderating effects of risk-taking propensity on the association between PTSD symptom severity and alcohol use in US veterans (Hawn et al., 2019). Risk-taking propensity was measured using the Balloon Analogue Risk Task, a laboratory-based behavioural measure where actions with a certain amount of risk yield a reward, but if risky behaviour is maintained it results in poorer outcomes (Lejuez et al., 2002). Hawn et al. (2019) found that risk-taking propensity did not moderate the relationship between PTSD and alcohol use (either weekly consumption or binge-drinking days, in the previous month).

#### 3.1.5. Quality assessment

Using the AXIS tool, the quality scores ranged from 9 to 19 points (out of a total score of 20 points), with nine studies scoring equal to or above 14 points (Table 3). All studies had clear aims, well-defined populations, validated outcome measures, appropriate reporting of statistical significance, and documented ethical approval or consent. The major issues noted across the studies were as follows. There was a lack of sample size justification, with only one study performing a power analysis (Karlović et al., 2012). Of the 13 cross-sectional studies, only one study was considered free from selection bias (Sartor et al., 2011). Seven studies had an all-male sample with PTSD participants (Karlović et al., 2004, 2012; Kim et al., 2013; Pennington et al., 2014; Sanjuan et al., 2013; Starčević et al., 2015; Young et al., 2002). One study specifically excluded female participants (Starčević et al., 2015). In five studies there was no statement regarding whether the study employed consecutive sampling (Karlović et al., 2004, 2012; Kim et al., 2013; Pennington et al., 2014; Young et al., 2002). Sanjuan, Thoma et al. (2013) initially recruited both sexes but ultimately excluded females from the analysis owing to a low sample size (and known sex differences). The authors appropriately specified males in the study title, yet the final sample still did not accurately reflect the broader PTSD & AUD population owing to strict exclusion criteria, such as left-handedness, active suicidal ideation, or claustrophobia. In addition, eight of the 13 cross-sectional studies excluded participants for current psychoactive medication use (Brady, Back, et al., 2006; Brady, Waldrop, et al., 2006; McRae et al., 2006; Danielson et al., 2021; Hawn et al., 2019; Pennington et al., 2014; Sanjuan et al., 2013; Starčević et al., 2015).

**Table 3.** Quality assessment of studies using the AXIS tool.

Study	Q1	Q2	Q3	Q4	Q5	Q6	Q7	Q8	Q9	Q10	Q11	Q12	Q13	Q14	Q15	Q16	Q17	Q18	Q19	Q20	Overall
Pennington et al. (2014)	Y	Y	N	Y	Y	N	N/A	Y	Y	Y	Y	Y	Unsure	N/A	Y	N	Y	Y	N	Y	14/20
Danielson et al. (2021)	Y	Y	N	Y	Y	N	N	N	Y	Y	Y	Y	Y	N	Y	Y	Y	Y	Unsure	Y	13/20
Brady, Back, et al. (2006)	Y	Y	N	Y	Y	N	N	Y	Y	Y	Y	Y	Y	N	Y	N	Y	Y	N	Y	14/20
Young et al. (2002)	Y	Y	N	Y	Y	Unsure	N/A	Y	Y	Y	Y	Y	Unsure	N/A	Y	Y	Y	Y	N	Y	15/20
Karlović et al. (2004)	Y	Y	N	Y	N	N	Y	Y	Y	Y	Y	Y	N	Y	Y	N	Y	N	Unsure	Y	14/20
Sanjuan et al. (2013)	Y	Y	N	Y	Y	N	Y	Y	Y	Y	Y	Y	N	Y	Y	Y	Y	Y	N	Y	18/20
Karlović et al. (2012)	Y	Y	Y	Y	Unsure	N	N	N	Y	Y	Y	Y	Unsure	Unsure	Y	Y	Y	N	Unsure	Y	12/20
McRae et al. (2006)	Y	N	N	Y	Y	N	N	Y	Y	Y	Y	N	Y	N	N	N	N	Y	N	Y	10/20
Brady, Waldrop, et al. (2006)	Y	Y	N	Y	Y	N	N	Y	Y	Y	Y	Y	Y	N	Y	Y	Y	Y	N	Y	15/20
Hawn et al. (2019)	Y	Y	N	Y	Y	N	N	Y	Y	Y	Y	Y	Unsure	N/A	Y	Y	Y	Y	N	Y	15/20
Kim et al. (2013)	Y	Y	N	Y	N	N	N	Y	Y	Y	Y	Y	Unsure	N/A	Y	Y	Y	Y	N	Y	14/20
Starčević et al. (2015)	Y	Y	N	Y	N	N	N	N	Y	Y	N	N	Unsure	N/A	Y	N	Y	N	N	Y	9/20
Sartor et al. (2011)	Y	Y	N	Y	Y	Y	Y	Y	Y	Y	Y	Y	N	Y	Y	Y	Y	Y	N	Y	19/20

Note: AXIS = Appraisal tool of Cross-Sectional Studies:

(Q1. Were the aims/objectives of the study clear?

(Q2. Was the study design appropriate for the stated aim(s)?

(Q3. Was the sample size justified?

(Q4. Was the target/reference population clearly defined? (Is it clear who the research was about?)

(Q5. Was the sample frame taken from an appropriate population base so that it closely represented the target/reference population under investigation?

(Q6. Was the selection process likely to select subjects/participants that were representative of the target/reference population under investigation?

(Q7. Were measures undertaken to address and categorize non-responders?

(Q8. Were the risk factor and outcome variables measured appropriate to the aims of the study?

(Q9. Were the risk factor and outcome variables measured correctly using instruments/measurements that had been trialled, piloted or published previously?

(Q10. Is it clear what was used to determined statistical significance and/or precision estimates? (e.g. *p* values, CIs)

(Q11. Were the methods (including statistical methods) sufficiently described to enable them to be repeated?

(Q12. Were the basic data adequately described?

(Q13. Does the response rate raise concerns about non-response bias? *(REVERSE CODED)*

(Q14. If appropriate, was information about non-responders described?

(Q15. Were the results internally consistent?

(Q16. Were the results for the analyses described in the methods, presented?

(Q17. Were the authors’ discussions and conclusions justified by the results?

(Q18. Were the limitations of the study discussed?

(Q19. Were there any funding sources or conflicts of interest that may affect the authors’ interpretation of the results? *(REVERSE CODED)*

(Q20. Was ethical approval or consent of participants attained?

Using the NOS tool, the quality assessment for the study by Kim et al. (2024) scored 8 stars (out of 9 stars). In accordance with the eligibility criteria, the study used CAPS-5 to assess PTSD, and AUD was assessed by heavy drinking. The main limitation of this study was that it was not clear whether PTSD was present at the intake assessment. Full scoring is provided in the Supplementary Results.

## 4. Discussion

While current evidence remains limited, this review of biological and neurocognitive mechanisms underlying comorbid PTSD & AUD identifies several promising directions. The main findings are: (1) despite having been extensively investigated, HPA axis dysregulation does not appear to be amplified in comorbid PTSD & AUD compared to PTSD only or AUD only; (2) certain genetic variants (i.e. *DRD2* and *APOE*) may be particularly relevant to individuals with PTSD & AUD; and (3) reduced hippocampal volume may represent a compounded impact of PTSD and AUD. Overall, further research is needed to clarify these mechanisms, particularly across more diverse samples that vary by sex and trauma type.

Baseline and stress-induced HPA axis function was a common thread across four studies (Brady, Back, et al., 2006; Brady, Waldrop, et al., 2006; Danielson et al., 2021; McRae et al., 2006). Despite hypotheses that there would be a cumulative impact of dysregulation of each condition in the comorbid group, this was not supported. This finding was apparent in a population of individuals with PTSD & AUD (Brady, Back, et al., 2006; Brady, Waldrop, et al., 2006) and in a cohort with PTSD and engaging in heavy drinking (Danielson et al., 2021). Each study used plasma to measure cortisol and ACTH, which are routinely outlined as difficult to measure as they are influenced by many factors, including diurnal rhythm (Weitzman et al., 1971), acute stress (i.e. social stress) (Figueiredo et al., 2020), medications/supplements (Cinar et al., 2008; Pedersen et al., 2023; Subramaniam et al., 2019), exercise habits (Caplin et al., 2021), diet (Tomiyama et al., 2010), recent infections (Alzahrani et al., 2021), and alcohol, including recency of use and family history (for a review, see Blaine & Sinha, 2017). A study by Forkus et al. (2024) using hair cortisol found that women with PTSD with high levels of cortisol had spent an additional 7.4 days drinking and 8.1 days binge drinking in the past 30 days compared to women with low cortisol. This study was not included in the review as participants were polysubstance users. Nonetheless, this paper highlights that cortisol plays a role in drinking behaviours in women with PTSD who use substances, and research in a population without concurrent substance use is necessary. Abnormalities in this neuroendocrine system have been found in PTSD (Metz et al., 2020; Morris et al., 2012; Yehuda et al., 1995), AUD (Bernardy et al., 1996; Errico et al., 1993; Sinha et al., 2009), and PTSD & AUD (Brady, Waldrop, et al., 2006; Forkus et al., 2024). The overlap in HPA axis may constitute a neurobiological mechanism underlying these three disorders. Notably, these studies did not report power calculations, and the combination of small sample sizes and substantial measurement variability may account for the absence of findings. To disentangle the exact role of the HPA axis in PTSD & AUD, future research may benefit from repeated measurements, or from using more practical sampling methods (e.g. saliva) (see review by El-Farhan et al., 2017) or scalp hair cortisol Wright et al., 2015).

There is substantial evidence of the shared genetic heritability between PTSD and substance use disorders (Koenen et al., 2003; Scherrer et al., 2008; Xian et al., 2000; Xie et al., 2009, 2010), and Sartor et al. (2011) extended these findings in the specific PTSD & AUD population. This review identified two candidate genes (*DRD2* and *APOE*) associated with PTSD & AUD. Specifically, Young et al. (2002) found increased *DRD2* A1 allelic frequency in male veterans with PTSD & AUD, complementing earlier findings by Comings et al. (1996) in PTSD and substance use disorder. Beyond PTSD, the *DRD2* gene is strongly implicated in addiction and other psychiatric conditions (see review by Noble, 2000). Importantly, the *DRD2* gene encodes the D_2_ dopamine receptor, and is associated with a variety of phenotypes, including working memory, emotion, and reward processing (Bertolino et al., 2010; Blasi et al., 2009, 2015; Di Giorgio et al., 2014; Peciña et al., 2013). Regarding *APOE*, Kim et al. (2013) found that male veterans who drank hazardously and were non-carriers of the *APOE* ϵ2 allele had a significantly increased risk of developing PTSD. *APOE* plays an critical role in the metabolism of lipoproteins and cholesterol (Mahley, 1988), and in neuronal repair and remodelling (Weisgraber & Mahley, 1996). Variants of *APOE* have been linked to a range of neurological and psychiatric conditions (for a review, see Forero et al., 2018), including depression (López-León et al., 2008), schizophrenia (González-Castro et al., 2015), frontotemporal dementia (Verpillat et al., 2002), PTSD (Freeman et al., 2005), and, most prominently, Alzheimer’s disease (Poirier et al., 1993). *APOE* has also been shown to be associated with the HPA axis in both clinical and preclinical studies (de Kloet et al., 2002; Grootendorst et al., 2002, 2004; Peavy et al., 2007). Although *DRD2* and *APOE* highlight promising genetic mechanisms, it is unlikely that variation in these genes alone explains the prevalence of PTSD & AUD, given their involvement in many other conditions. A major caveat of Young et al. (2002) and Kim et al. (2013) is that these are relatively small candidate-gene studies, which have demonstrated poor replicability across psychiatric genetics research (Border et al., 2019). In addition, PTSD and AUD are highly polygenic, with risk being distributed across a variety of loci (Kranzler et al., 2019; Nievergelt et al., 2018). Accordingly, *DRD2* and *APOE* may be implicated in co-occurring PTSD & AUD but are unlikely to independently drive the risk of PTSD & AUD. Future research should employ genome-wide association approaches to capture the broader polygenic architecture of this comorbidity.

Lower hippocampal volume in veterans with comorbid PTSD and AUD was observed in one study, and represents a relevant, although preliminary, finding from this review. This elongated subcortical structure, located in the medial temporal lobe, is central to learning and memory (Anderson et al., 2014; Nakazawa et al., 2004) and has consistently been identified as a region of interest independently in PTSD and AUD in studies using large pooled datasets (Logue et al., 2018; Mackey et al., 2019). Preclinical studies show that stress and alcohol reduce pyramidal neurons (Conrad et al., 1999; Lukoyanov et al., 2000) and suppress neurogenesis (Czéh et al., 2007; Malberg & Duman, 2003; Morris et al., 2010) in this region, probably mediated by dysregulation of the HPA axis and glucocorticoids (for reviews, see Kim et al., 2015; Stephens & Wand, 2012). Despite this, clinical findings in PTSD & AUD remain inconsistent. Schuff et al. (2008) found no hippocampal volume differences among PTSD & AUD, PTSD, AUD, or controls. In contrast, Logue et al. (2018) showed that hippocampal reductions in PTSD remained significant after controlling for AUD, suggesting that alcohol was not driving the effect. Importantly, Logue et al. (2018) and Schuff et al. (2008) did not differentiate between lifetime and current AUD. In contrast, Woodward et al. (2006) reported lower hippocampal volume in PTSD with lifetime AUD than in PTSD only, although current AUD was excluded. Complementing these studies, Starčević et al. (2015) showed lower bilateral hippocampal volume in PTSD with current AUD compared to PTSD and controls. Taken together, these findings indicate that hippocampal alterations are evident across both PTSD and AUD, with the possibility of compounded effects in their comorbidity. Importantly, the findings from a large population-based adolescent cohort (ABCD study) suggest that comorbidity is unlikely to reflect a simple additive neurobiological burden, instead pointing to distinct neurodevelopmental risk processes (Czéh et al., 2007). This underscores the need to determine whether volumetric alterations reflect shared vulnerability, disorder-specific adaptation, or features unique to comorbidity. Large-scale neuroimaging datasets, such as ENIGMA-PTSD, provide an opportunity to test these questions at scale, as well as to evaluate other neuroimaging findings reviewed here.

### 4.1. Strengths and limitations

This article is the first to systematically review the biological and cognitive mechanisms underpinning comorbid PTSD & AUD. Key strengths include the use of clinical diagnosis of PTSD, a preregistered protocol, risk of bias assessment, and the use of five databases. Limitations should also be noted. Most included studies recruited male and/or veteran samples, restricting generalizability. Alcohol-related conditions were also inconsistently defined [AUD (DSM-5), alcohol abuse/dependence (DSM-IV), hazardous drinking (WHO), heavy drinking (NIAAA)], introducing heterogeneity. This broad operationalization may have influenced findings, as it does not adequately account for differences in severity, dependence, or alcohol-related harm, which can contribute to variability in biological mechanisms and risk (Al-Khalil et al., 2021; Kranzler et al., 2019). Notably, evidence indicates that cortisol alterations may be driven more by current alcohol consumption patterns than by diagnostic status alone (Boschloo et al., 2011), suggesting that collapsing alcohol-related conditions may obscure meaningful biological differences. The literature remains sparse: cortisol was the only measure assessed across multiple cohorts, with no consistent associations (Brady, Back, et al., 2006; Brady, Waldrop, et al., 2006; Danielson et al., 2021; McRae et al., 2006). Genetic evidence also remains limited. Despite large-scale studies demonstrating overlap between PTSD and AUD, only the study by Sartor et al. (2011) met the inclusion criteria; others were excluded for using existing summary statistics for each independent disorder (Bountress, Brick, et al., 2022; Bountress, Bustamante, et al., 2022), examining substance use disorder broadly (Koenen et al., 2003; Scherrer et al., 2008; Xian et al., 2000; Xie et al., 2009, 2010), or not assessing comorbidity (Bountress et al., 2021; Saenz de Viteri et al., 2023).

### 4.2. Conclusion

This review highlights neurobiological and genetic alterations that may be distinct to co-occurring PTSD & AUD, although further research is required to confirm these findings. A consistent theme is HPA axis dysregulation, which, despite existing hypotheses, does not appear to be amplified in PTSD & AUD, potentially as a result of methodological limitations in sample collection or sample size. Given its high prevalence and adverse outcomes, there is an urgent need for research directly examining the molecular, genetic, neural, and cognitive pathways involved in PTSD & AUD. Advancing our understanding of PTSD & AUD biology may not only clarify its underlying mechanisms but also inform the development of targeted, mechanism-based interventions. Future studies should use existing neuroimaging and genetic datasets to more precisely characterize the mechanisms underlying this comorbidity.

## Supplementary Material

SupplementaryInformation_040126.docx

Customised_Data_Extraction_Sheet.xlsx

## Data Availability

Data sharing is not applicable to this article as no datasets were generated or analysed during the current study.
