# Supplementary material for: Biological and neurocognitive correlates of comorbid post-traumatic stress disorder and alcohol use disorder: a systematic review
Source: Eur J Psychotraumatol. 2026 May 26;17(1):2640736. doi: 10.1080/20008066.2026.2640736 (PMC13215429; doi:10.1080/20008066.2026.2640736)
Supplement: SupplementaryInformation_040126.docx [file ZEPT_A_2640736_SM6364.docx]

# Supplementary Information

# Methods

## Data Extraction

For each biomarker domain - molecular, genetic, neural and cognitive - additional methodological and result information were collected. Studies examining molecular correlates provided data on; (1) the type of biomarker measured, for instance, cortisol or BDNF, (2) if a concurrent task was use, (3) the measurement method employed such as ELISA or radioimmunoassay, (4) the time of sample collection, (5) the unit of measurement (ng/dL or nmol/L). For studies involving genetic information, data extracted comprised; (1) the genotyping method used (such as PCR or sequencing), (2) the target gene and the (3) the separation method, (4) mechanism of gene.

For neuroimaging studies, extracted data included (1) the imaging modality (for example, structural or resting-state), (2) scanner and acquisition details, (3) type of analysis performed (such as region of interest (ROI) or whole-brain analysis) and the seeds used if relevant, (4) any relevant metrics (i.e., neurometabolites, BOLD signals, or T1-weighted images), (5) image processing techniques. Lastly, for studies examining cognition, information extracted encompassed; (1) the type of cognitive domain assessed (such as memory or executive functioning), (2) the specific tests (like the Stroop Test or Wisconsin Card Sorting Test), and (3) details of test administration.

## SEARCH STRATEGY

| **Database** | **Search Terms** |
| --- | --- |
| **EMBASE**  (2159) | (‘posttraumatic stress disorder’[MeSH] OR ‘ptsd’:ti,ab,kw OR ‘ptss’:ti,ab,kw OR ‘stress disorder’;ti,ab,kw OR ‘post-traumatic stress disorder’:ti,ab,kw OR ‘post trauma*’:ti,ab,kw OR ‘post-trauma*’:ti,ab,kw OR ‘posttrauma*’:ti,ab,kw)  AND  (‘alcoholism’ [MeSH] OR ‘AUD’:ti,ab,kw OR ‘alcohol depend*’:ti,ab,kw OR ‘alcohol use disorder’:ti,ab,kw OR ‘heavy drink*’:ti,ab,kw OR ‘harmful drink*’:ti,ab,kw)  AND  (‘neuroimaging’[MeSH] OR ‘nuclear magnetic resonance imaging’[MeSH] OR ‘functional neuroimaging’[MeSH] OR ‘dopaminergic imaging’[MeSH] OR ‘magnetic resonance imaging’:ti,ab,kw OR ‘brain scan*’:ti,ab,kw OR ‘fMRI’:ti,ab,kw OR ‘functional magnetic resonance imaging’:ti,ab,kw OR ‘nuclear magnetic resonance’[MeSH] OR ‘MRS’:ti,ab,kw OR ‘nuclear magnetic resonance spectroscopy’[MeSH] OR ‘magnetic resonance spectroscopy’:ti,ab,kw OR ‘positron emission tomography’[MeSH] OR ‘PET’:ti,ab,kw OR ‘neurocircuit*’:ti,ab,kw OR ‘functional connectivity’[MeSH] OR ‘functional connectivity’:ti,ab,kw OR ‘gene’[MeSH] OR ‘gene*’:ti,ab,kw OR ‘dna’[MeSH] OR ‘DNA’:ti,ab,kw OR ‘methylation’[MeSH] OR ‘methylat*’:ti,ab,kw OR ‘epigenetics’[MeSH] OR ‘epigene*’:ti,ab,kw OR ‘hormon*’:ti,ab,kw OR ‘cortisol’:ti,ab,kw OR ‘hormone blood level’[MeSH] OR ‘hormone release’[MeSH] OR ‘oxytocin’:ti,ab,kw OR ‘endocrine function’[MeSH] OR ‘hpa’:ti,ab,kw OR ‘HPA axis’:ti,ab,kw OR ‘hypothalamic-pituitary-adrenal axis’:ti,ab,kw OR ‘brain derived neurotrophic factor’[MeSH] OR ‘BDNF’:ti,ab,kw OR ‘brain derived neurotropic factor’:ti,ab,kw OR ‘*cognitive assessment*’:ti,ab,kw OR ‘*cognitive test*’:ti,ab,kw OR ‘*cognitive examination*’:ti,ab,kw OR ‘*cognitive evaluation’:ti,ab,kw OR ‘corticotrophin’[MeSH] OR ‘corticotropin’:ti,ab,kw OR ‘acth’:ti,ab,kw OR ‘corticotrophin releasing factor’[MeSH] OR ‘corticotropin releasing factor’:ti,ab,kw) |
| **SCOPUS**  (2052) | ( TITLE-ABS-KEY ( ( "posttraumatic stress disorder" OR "ptsd" OR "ptss" OR "stress disorder" OR "post-traumatic stress disorder" OR "post trauma*" OR "post-trauma*" OR "posttrauma*" ) ) AND TITLE-ABS-KEY ( "alcoholism" OR "AUD" OR "alcohol depend*" OR "alcohol use disorder" OR "heavy drink*" OR "harmful drink*" ) AND TITLE-ABS-KEY ( "neuroimaging" OR "functional neuroimaging" OR "dopaminergic imaging" OR "magnetic resonance imaging" OR "brain scan*" OR "fMRI" OR "functional magnetic resonance imaging" OR "nuclear magnetic resonance*" OR "MRS" OR "magnetic resonance*" OR "positron emission tomography" OR "PET" OR "neurocircuit*" OR "functional connectivity" OR "gene*" OR "dna" OR "DNA" OR "methylated dna*" OR "methylat*" OR "epigene*" OR "hormon*" OR "cortisol" OR "oxytocin" OR "hormone blood" OR "hormone release" OR "hormone receptor" OR "endocrine function" OR "hpa*" OR "hypothalamic-pituitary-adrenal axis" OR "brain derived neurotrophic factor" OR "BDNF" OR "cognitive assessment*" OR "neurocognitive assessment*" OR "cognitive test*" OR "neurocognitive test*" OR "cognitive examination*" OR "neurocognitive examination*" OR "cognitive evaluation" OR "neurocognitive evaluation" OR corticotropin OR acth OR "corticotropin releasing factor" )) |
| **APA PsychInfo** (520) | (exp ‘Posttraumatic Stress Disorder’[MeSH] OR ‘posttraumatic stress disorder’:ti,ab,kw OR exp ‘Posttraumatic Stress’[MeSH] OR ‘ptss’:ti,ab,kw OR ‘stress disorder’:ti,ab,kw OR ‘post trauma*’:ti,ab,kw OR ‘post-trauma*’:ti,ab,kw OR ‘posttrauma’:ti,ab,kw)  AND  (exp ‘alcoholism’[MeSH] OR ‘AUD’:ti,ab,kw OR ‘alcohol depend*’:ti,ab,kw OR ‘alcohol use disorder’:ti,ab,kw OR ‘heavy drink*’:ti,ab,kw or ‘harmful drink*’:ti,ab,kw)  AND  (exp ‘neuroimaging’[MeSH] OR exp ‘nuclear magnetic resonance imaging’ [MeSH] OR exp ‘functional neuroimaging’ [MeSH] OR exp ‘dopaminergic imaging’ [MeSH] OR ‘magnetic resonance imagin’:ti,ab,kw OR ‘brain scan*’:ti,ab,kw OR ‘fMRI’:ti,ab,kw OR ‘functional magnetic resonance imaging’:ti,ab,kw OR exp nuclear magnetic resonance’ [MeSH] OR ‘MRS’:ti,ab,kw OR exp ‘nuclear magnetic resonance spectroscopy’ [MeSH] OR ‘magnetic resonance spectroscopy’:ti,ab,kw OR exp ‘positron emission tomography’ [MeSH] OR ‘PET’:ti,ab,kw OR ‘positron emission tomography’:ti,ab,kw OR ‘neurocircuit*:ti,ab,kw OR exp ‘functional connectivity’ [MeSH] OR ‘functional connectivity’:ti,ab,kw OR exp ‘gene’ [MeSH] OR ‘gene’:ti,ab,kw OR exp ‘dna’ [MeSH] OR ‘DNA’:ti,ab,kw OR exp ‘methylation’ [MeSH] OR ‘methylat*’:ti,ab,kw OR exp ‘epigenetics’ [MeSH] OR ‘epigene*’:ti,ab,kw OR ‘hormon*’:ti,ab,kw OR ‘cortisol’:ti,ab,kw OR exp ‘hormone blood level’ [MeSH] OR exp ‘hormone release’ [MeSH] OR ‘oxytocin’:ti,ab,kw OR exp ‘endocrine function’ [MeSH] OR ‘hpa’:ti,ab,kw OR ‘HPA axis’:ti,ab,kw OR ‘hypothalamic-pituitary-adrenal axis’:ti,ab,kw OR exp ‘brain derived neurotrophic factor’ [MeSH] OR ‘BDNF’:ti,ab,kw OR ‘brain derived neurotrophic factor’:ti,ab,kw OR ‘*cognitive assessment*’:ti,ab,kw OR ‘*cognitive test*’:ti,ab,kw OR ‘*cognitive examination*’:ti,ab,kw OR ‘*cognitive evaluation’:ti,ab,kw OR exp ‘corticotropin’ [MeSH] OR ‘corticotropin’:ti,ab,kw OR ‘acth’:ti,ab,kw OR exp ‘corticotropin releasing factor’ [MeSH] OR ‘corticotropin releasing factor’:ti,ab,kw) |
| **MEDLINE via OVID**  (646) | (‘stress disorders, post-traumatic’ [MeSH] OR ‘stress disorders, traumatic, acute’ [MeSH] OR ‘ptsd’:ab,ti,kw OR ‘ptss’:ti,ab,kw OR ‘stress disorder’:ti,ab,kw OR ‘post-traumatic stress disorder’:ti,ab,kw OR ‘post trauma*’:ti,ab,kw OR ‘post-trauma*’:ti,ab,kw OR ‘posttrauma*’:ti,ab,kw)  AND  (‘alcohol-related disorders’ [MeSH] OR ‘alcoholism’ [MeSH] OR ‘binge drinking’ [MeSH] OR ‘AUD’:ti,ab,kw OR ‘alcohol depend*’:ti,ab,kw OR ‘alcohol use disorder’:ti,ab,kw OR ‘heavy drink*’:ti,ab,kw OR ‘harmful drink*’:ti,ab,kw)  AND  (‘Neuroimaging’ [MeSH] OR ‘Functional Neuroimaging’ [MeSH] OR ‘Positron-Emission Tomography’ [MeSH] OR ‘Dopaminergic Imaging’ [MeSH]  OR ‘magnetic resonance imaging’:ti,ab,kw OR ‘brain scan*’:ti,ab,kw OR ‘fMRI’:ti,ab,kw OR ‘functional magnetic resonance imaging’:ti,ab,kw OR ‘MRS’:ti,ab,kw OR ‘Magnetic Resonance Spectroscopy’ [MeSH] OR ‘magnetic resonance spectroscopy’:ti,ab,kw OR ‘PET’:ti,ab,kw OR ‘positron emission tomography’:ti,ab,kw OR ‘neurocircuit*’:ti,ab,kw OR ‘functional connectivity’:ti,ab,kw OR ‘Genes’ [MeSH] OR ‘gene*’:ti,ab,kw OR ‘DNA’ [MeSH] OR ‘Methylation’ [MeSH] OR ‘DNA Methylation’ [MeSH] OR ‘methylat*’:ti,ab,kw OR ‘Epigenomics’ [MeSH] OR ‘epigene*’:ti,ab,kw OR ‘hormon*’:ti,ab,kw OR ‘cortisol’:ti,ab,kw OR ‘Hormones’ [MeSH] OR ‘oxytocin’:ti,ab,kw OR ‘hpa’:ti,ab,kw OR ‘HPA axis’:ti,ab,kw OR ‘hypothalamic-pituitary-adrenal axis’:ti,ab,kw OR ‘Brain-Derived Neurotrophic Factor’ [MeSH] OR ‘brain derived neurotrophic factor’:ti,ab,kw OR ‘BNDF’:ti,ab,kw OR ‘brain derived neurotrophic factor’:ti,ab,kw OR ‘*cognitive assessment*’:ti,ab,kw OR ‘*cognitive test*’:ti,ab,kw OR ‘*cognitive examination*’:ti,ab,kw OR ‘*cognitive evaluation’:ti,ab,kw OR ‘Neuropsychological Tests’ [MeSH] OR ‘Adrenocorticotropic Hormone’ [MeSH] OR ‘corticotropin’:ti,ab,kw OR ‘acth’:ti,ab,kw OR ‘Corticotropin-Releasing Hormone’ [MeSH] OR ‘corticotropin releasing factor’:ti,ab,kw) |
| **Web of Science**  (1492) | ((ALL=("posttraumatic stress disorder" OR "ptsd" OR "ptss" OR "stress disorder" OR "post-traumatic stress disorder" OR "post trauma*" OR "post-trauma*" OR "posttrauma*" ))  AND  ALL=("alcoholism" OR "AUD" OR "alcohol depend*" OR "alcohol use disorder" OR "heavy drink*" OR "harmful drink*" )) AND ALL=("neuroimaging" OR "functional neuroimaging" OR "dopaminergic imaging" OR "magnetic resonance imaging" OR "brain scan*" OR "fMRI" OR "functional magnetic resonance imaging" OR "nuclear magnetic resonance*" OR "MRS" OR "magnetic resonance*" OR "positron emission tomography" OR "PET" OR "neurocircuit*" OR "functional connectivity" OR "gene*" OR "dna" OR "DNA" OR "methylated dna*" OR "methylat*" OR "epigene*" OR "hormon*" OR "cortisol" OR "oxytocin" OR "hormone blood" OR "hormone release" OR "hormone receptor" OR "endocrine function" OR "hpa*" OR "hypothalamic-pituitary-adrenal axis" OR "brain derived neurotrophic factor" OR "BDNF" OR "cognitive assessment*" OR "neurocognitive assessment*" OR "cognitive test*" OR "neurocognitive test*" OR "cognitive examination*" OR "neurocognitive examination*" OR "cognitive evaluation" OR "neurocognitive evaluation" OR corticotropin OR acth OR "corticotropin releasing factor") |

Acronym: ti,ab,kw = title, abstract, keywords; MeSH = Medical Subject Heading.

**NEWCASTLE - OTTAWA QUALITY ASSESSMENT SCALE**

**COHORT STUDIES (Wells et al., 2000)**

**(Kim et al., 2024)**

Note: A study can be awarded a maximum of one star for each numbered item within the Selection and Outcome categories. A maximum of two stars can be given for Comparability

**Selection**

1) Representativeness of the exposed cohort

a) truly representative of the average _______________ (describe) in the community

**b) somewhat representative of the average PTSD in the community 🟑**

c) selected group of users eg nurses, volunteers

d) no description of the derivation of the cohort

2) Selection of the non exposed cohort

**a) drawn from the same community as the exposed cohort 🟑**

b) drawn from a different source

c) no description of the derivation of the non exposed cohort

3) Ascertainment of exposure

**a) secure record (eg recruitment from hospital) 🟑**

b) structured interview

c) written self report

d) no description

4) Demonstration that outcome of interest was not present at start of study

a) yes

**b) no**

**Comparability**

1) Comparability of cohorts on the basis of the design or analysis

a) study controls for **PTSD** (select the most important factor) **🟑**

b) study controls for any additional factor  **🟑** **age, sex, prior psychiatric history, unemployment status, previous traumatic events, number of physical disorders, body mass index, and scores on Connor-Davidson Resilience Scale, Injury Severity Score, and Hospital Anxiety and Depression Scale-depression subscale.**

**Outcome**

1) Assessment of outcome

a) independent blind assessment  **🟑** (structured clinical interview)

b) record linkage

c) self report

d) no description

2) Was follow-up long enough for outcomes to occur

a) yes (select an adequate follow up period for outcome of interest) **🟑** (3, 6, 12, 24 months post exposure)

b) no

3) Adequacy of follow up of cohorts

a) complete follow up - all subjects accounted for

b) subjects lost to follow up unlikely to introduce bias - small number lost **18.463%** (select an adequate %) follow up, or description provided of those lost) **🟑**

c) follow up rate < ____% (select an adequate %) and no description of those lost

d) no statement

# References

Kim, J. M., Kang, H. J., Kim, J. W., Jang, H., Kim, J. C., Lee, J. Y., Kim, S. W., & Shin, I. S. (2024). Delayed effects of alcohol consumption on the association between serum BDNF levels and post-traumatic stress disorder development over two-years [Article]. *Progress in Neuro-Psychopharmacology and Biological Psychiatry*, *135*, Article 111106. <https://doi.org/10.1016/j.pnpbp.2024.111106>

Wells, G. A., Shea, B., O’Connell, D., Peterson, J., Welch, V., Losos, M., & Tugwell, P. (2000). The Newcastle-Ottawa Scale (NOS) for assessing the quality of nonrandomised studies in meta-analyses.
